# Stemming the Wave of Cervical Cancer: Human Papillomavirus Vaccine Introduction in India

**DOI:** 10.1200/JGO.17.00030

**Published:** 2017-09-08

**Authors:** Ravi Mehrotra, Roopa Hariprasad, Preetha Rajaraman, Vini Mahajan, Rajesh Grover, Prabhdeep Kaur, Soumya Swaminathan

**Affiliations:** **Ravi Mehrotra** and **Roopa Hariprasad**, National Institute of Cancer Prevention and Research, Noida; **Preetha Rajaraman**, Center for Global Health, National Cancer Institute; **Rajesh Grover**, Delhi State Cancer Institute; **Soumya Swaminathan**, Indian Council of Medical Research, New Delhi; **Vini Mahajan**, Health and Family Welfare, Chandigarh, Punjab; and **Prabhdeep Kaur**, National Institute of Epidemiology, Chennai, India.

With the highest burden of cervical cancer mortality in the world, India faces enormous loss of life, productivity, and suffering from this disease. The age-standardized incidence ratio for cervical cancer in India is 22 per 100,000 women per year, which is higher than the incidence in South East Asia (16.3 per 100,000) and in the world (14 per 100,000).^1^ Differences in mortality rates are even starker. Twenty-five percent of all global deaths as a result of cervical cancer occur in India.^1^ Although cervical cancer rates in India are decreasing, there is huge geographic variability, and the absolute number of patients is still expected to increase over the next decade as a result of population growth.^1,2^ Moreover, the decreasing incidence rate seems to be plateauing and is unlikely to decline further unless specific interventions are put in place.^3^

Although widespread and organized screening programs can facilitate earlier detection and management of precancers and cancers, thereby leading to decreased mortality, such screening programs are logistically highly complex, resource intensive, and at a nascent stage in India. Less than 5% of the eligible women in India have ever been screened.^4^ Recently, a screening program has been recommended by the government that involves screening women every 5 years using visual inspection by acetic acid as the primary mode of screening.^5^ With proper implementation, this screening program would begin to address the prevention of cervical cancer. However, it is imperative that along with this secondary prevention program, there is a strong focus on primary prevention.

Globally, it is now widely accepted that vaccination against high-risk strains of the human papillomavirus (HPV) is a safe and effective means of primary prevention of cervical cancer. More than 80 countries have introduced HPV vaccination in their national immunization programs, of which 33 are low- and middle-income countries (LMICs).^3^ Cost-effectiveness studies on HPV vaccination have shown that spending on HPV vaccinations is more cost effective than treating cervical cancer.^6^

Although HPV vaccines have been licensed for use in India by the Drug Controller General of India since 2008, until recently, these vaccines have not been included in the routine government immunization program. The issue of HPV vaccine introduction into government programs has been the subject of debate among academic, medical, public health, and advocacy groups; nongovernmental organizations; and parliament. Scientific publications have argued for and against vaccine introduction in India.^7,8^ A vaccine delivery and demonstration project led by an international nonprofit organization, PATH, was started in 2009 in Andhra Pradesh and Gujarat but was suspended in 2010 as a result of public concern, allegedly arising from the deaths of seven girls who received HPV vaccine.^9,10^ Subsequent investigations concluded that these deaths were not linked to vaccination but occurred as a result of varied unrelated causes such as snake bite, epilepsy, malaria, and suicide. Unfortunately, the project was never restarted but, collaterally, led to the development of more stringent regulatory, ethical, quality control, and monitoring standards of clinical trials in the country, as well as serendipitously provided proof for the efficacy of a two-dose regimen in the 9- to 13-year age group, which was later also approved by the WHO.^11^

The past few months have seen the beginning of the turning of the tide. The Indian Council of Medical Research (ICMR) organized multiple expert group meetings with various stakeholders to review the state of knowledge and current global recommendations regarding the HPV vaccine, as well as to prepare interested state governments for initiating this activity. The expert group endorsed the following recommendations for the introduction of HPV vaccine in the programmatic settings in India.

1. The experience of developed as well as developing countries around the world in the past 10 years and WHO recommendations are unequivocally in favor of introducing HPV vaccination in the government’s immunization program.
2. Girls age 9 to 13 years, translating to girls in grade 6, would form the target group for protection. Both government and private schools should be covered to ensure herd immunity. Two doses of the vaccine should be given at a gap of 6 to 12 months.
3. Both the bivalent and quadrivalent vaccines are equally effective in the Indian context, and price could be used as the selection criterion.
4. Vaccination should be accompanied by cervical cancer screening programs for mothers accompanying the female child.
5. Parents should be informed and their consent obtained with no element of compulsion in case of any unwilling family.
6. ICMR will assist in collecting evidence on HPV vaccine and developing a policy brief.
7. ICMR’s role in introduction of the program in states will be to monitor and document feasibility of program implementation.

Delhi was the first state in India to initiate a public HPV vaccination program for school children, on the occasion of National Cancer Awareness Day (November 7, 2016).^12^ This program invited girls age 11 to 13 years to get vaccinated at the Delhi State Cancer Institutes (East and West). A total of 1,200 doses have been administered as of March 2017 in a hospital setting along with mothers being offered the Papanicolaou smear and mammography services. No serious adverse events have been reported.

The Delhi government would like to expand the program to vaccinate all class 6 students through the school health program, which will include girls from both private and public schools numbering approximately 250,000 per annum.

On November 23, 2016, the government of Punjab also initiated HPV vaccination in a campaign in the Bathinda (incidence 17.5 per 100,000 women) and Mansa (17.3 per 100,000 women) districts. In phase 1, nearly 10,000 girls studying in class 6 of government schools were covered.^13^ A total of 261 schools in Bathinda and 187 schools in Mansa were involved in the program. In total, 5,851 girls were vaccinated at Bathinda and 4,002 at Mansa, constituting 97.5% and 98.5% coverage, respectively. Although 28 minor adverse events were reported, consisting of fainting (n = 16), dizziness (n = 5), vomiting (n = 5), and headache (n = 2), these were managed locally with complete recovery, without any need for referral to a higher center. In the second phase, plans are afoot to include five more districts, which have the next highest incidence rates of the disease, thereby covering all districts that have a reported incidence of > 10 per 100,000 women. The program will be gradually scaled up to include all girls in class 6 in both government and private schools across the state. The program is adopting both a facility-based and school-based approach to vaccination in the second phase.

These initial programs mark the first steps toward elimination of cervical cancer burden in India over the next decades. Along with vaccination, awareness regarding other primary preventive measures, such as tobacco cessation and safe sexual practices, should accompany cervical cancer control.^14^

Past studies have reported the challenges and barriers in LMICs, and lessons have been learned from the implementation of HPV vaccine rollout in many LMICs.^15,16^ The key barriers evidenced by this research were sociocultural, health systems, and financial barriers. However, progress has been made through financing mechanisms such as the Global Alliance for Vaccines and Immunization and other organizations that aim to improve access of eligible LMICs to vaccines through negotiating lower vaccine prices and cofunding until countries can afford the vaccines.^17^ The Global Alliance for Vaccines and Immunization is a global health partnership of public- and private-sector organizations that provides a unique opportunity for a wide range of partners to build consensus around policies, strategies, and priorities and to recommend responsibility of implementation to the partner with the most experience and insight in the area. These funding opportunities have opened the window for eligible countries to implement national programs for countries that already have experience with delivering the vaccine.

Given that these are the first large-scale HPV vaccination introduction programs in India, experiences gained at both the programmatic and the community level will be key to scaling up the program within the states and in other parts of the country. Although these primary prevention efforts are expected to have an enormous impact on the expected burden of cervical cancer in regions where they are implemented, the comprehensive control of cervical cancer will still require addressing all aspects of prevention and care, including increasing awareness, screening and early detection of cervical lesions, appropriate treatment, and palliative care when necessary.
